# Concept Maps in Immunology: A Metacognitive Tool to Promote Collaborative and Meaningful Learning among Undergraduate Medical Students

**DOI:** 10.30476/JAMP.2022.94275.1576

**Published:** 2022-07-01

**Authors:** MOHAN B SANNATHIMMAPPA, VINOD NAMBIAR, RAJEEV ARAVINDAKSHAN

**Affiliations:** 1 Department of Microbiology, College of Medicine and Health Sciences, National University of Science and Technology, Sohar Campus, Sultanate of Oman; 2 Department of community medicine, All India Institute of Medical Sciences, Mangalagiri, Andhra Pradesh, India

**Keywords:** Learning, Collaborative, Critical thinking, Knowledge, Metacognition

## Abstract

**Introduction:**

Concept maps are graphical representations of knowledge that connect concepts, ideas, and relationships. The present study aims at assessing the perception of medical students in utilization of concept maps as a tool to foster their lifelong learning skills in immunology.

**Methods:**

The current study was approved by Institutional Ethics and Review Committee. Third-year undergraduate (MD3) medical students of the academic year 2021-22 were sensitized about the concept map study and included after obtaining their informed consent. The students worked in teams to find answers and link the different words or phrases of the concept maps. At the end of the immunology course, students’ perception on concept map-based learning strategy was assessed. A pre-designed, self-administered questionnaire, pre-validated by subject experts for relevance and feasibility, was used for the study. The questionnaire included some quantitative questions assessed by using 3-point Likert Scale and an open-ended question to receive students’ comments on concept map-based learning strategy. The responses were collected and analyzed using SPSS version 22. Descriptive statistics was used for the quantitative variables, tabulated as numbers and percentages while the qualitative data was analyzed by thematic analysis. The quantitative data results were prioritized but supported by students’ comments on open ended question.

**Results:**

Out of 133 eligible participants, 109 students who volunteered and completed the study were included. Majority of our participants (>80%) welcomed the concept map-based learning strategy. Almost 4 out of 5 expressed that concept maps are interesting and enjoyable, encourage active participation, peer discussion, and enhance critical thinking and problem-solving skills. More than 80% of the students agreed that concept maps promoted deep understanding of the topic and lifelong learning. Nearly 3 out of 4 students suggested including concept maps in many immunology topics in future. Majority of students penned down positive comments indicating concept map tool facilitates metacognitive skills.

**Conclusion:**

From the study, it can be concluded that concept maps are effective active learning strategies to improve the metacognitive domain of medical students in immunology course, thus assisting them to become better lifelong learners.

## Introduction

**Figure 1:**
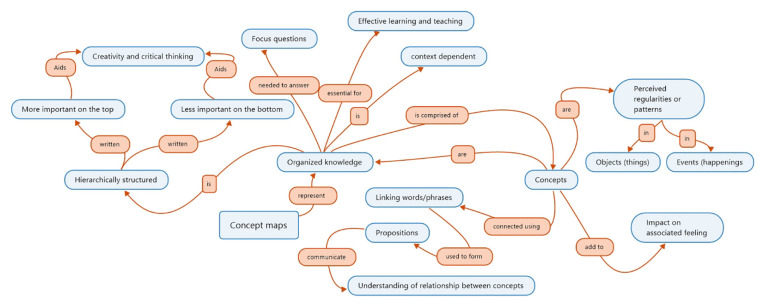
Illustration of core elements of the concept map

The highly competency-based modern medical education entails medical undergraduates to be contemplative to practice medicine in future ( 1 , 2 ). To emerge as better lifelong learners, medical undergraduates must possess essential qualities such as critical thinking, clinical reasoning, deep understanding of concepts, and ability to apply the gained knowledge to solve the problems ( 3 , 4 ). Traditional didactic delivery of medical curriculum involves little students’ participation and tends to promote rote memorization. Hence, medical educators in recent years have shown more inclination towards student-centered, active learning techniques ( 5 - 7 ). Active-learning strategies emphasize learners’ engagement, self-awareness of their learning process, and thus enhance lifelong learning skills ( 8 - 10 ). Concept mapping is relatively a new teaching-learning strategy introduced by Joseph Novak in 1970 and has been utilized by educators as a powerful education tool to promote active participation and nurture meaningful learning among students and also, as a tool for assessment ( 8 , 11 , 12 ). Concept maps are two-dimensional graphical tools for organizing and representing knowledge ( 8 ). Figure 1 represents core components of concept maps.

The concept maps include concepts usually enclosed within circles or boxes of some type with connecting lines linking two concepts ( 13 , 14 ). The relationship between two concepts is specified by words on the line, referred to as linking words or phrases. Concept maps support higher order thinking skills better than many other types of instructional strategies. Creating concept maps requires a clear understanding of how different concepts are related to one another and hence facilitates gaining better knowledge which cannot be achieved simply through rote learning or memorization. The relationship between concepts is expressed in the form of propositions and the propositions that make up a meaningful statement containing multiple concepts are called ‘semantic units’. These units are hierarchical usually with broader generalized concepts at the top of the concept maps and the specific ones at the bottom with strong propositions connecting the two concepts ( 15 ). Crosslinks can be formed to give answers to the ‘focus question’ ( 13 ). Concept maps provide several advantages for learners. Systematic and orderly arrangement of concepts facilitates students’ easy understanding of difficult concepts. It provides opportunities to students to make relevant and key connections within the body of knowledge, enhances interest, encourages generation of new ideas, and promotes one’s own critical thinking, creativity, solving complex problems, and integrating theory to practice. Further, it facilitates student collaboration, helps in identifying the gaps in learning, clarifies misconceptions or doubts, and assists in establishing a relationship between previous knowledge and newly introduced concepts, and thus, fosters meaningful learning rather than rote learning ( 11 , 15 ). Concept maps for learning are generally used in one of the following ways: students are asked to construct concept maps on their own on a focused topic or students are asked to analyze concept maps designed by the instructor on a specific topic and connect the terms or phrases. Both approaches are effective in enhancing the students’ learning outcomes ( 8 , 16 , 17 ). 

Immunology is one of the basic science subjects in medical education and it requires familiarization of terminologies, acquisition of critical thinking, clinical reasoning, and integrating different concepts for deep understanding and application of gained knowledge in solving the problem. It is a challenge for medical students to connect the concepts of immunology to understand immunological diseases ( 18 , 19 ). The concept maps are excellent supporting tools in immunology and benefit learners in many ways such as summarizing key topics, identifying the key areas that need to be focused, understanding concepts in depth, uncovering misconceptions, expressing ideas on complex conceptual framework, and as a self-assessment tool ( 20 ). The design of concept maps may vary from absolute freedom to students to construct concept maps on their own without providing any terms to more restrictive approaches where skeleton is provided, and students need to connect the terms or phrases ( 21 ). Having done thorough literature research, we could not find any studies on concept maps in medical education in Oman. Hence, the current study was aimed to introduce concept mapping in the immunology course and assess the students’ perception on the effectiveness of concept mapping in the immunology course in promoting their meaningful learning. 

## Methods

The present study was conducted in the Department of Microbiology and Immunology, College of Medicine and Health Sciences (CoMHS), National University. The Institutional Ethics and Review Committee approved the study (Approval no. NU/COMHS/EBC0028/2021). The study participants were third-year (MD3) undergraduate medical students (n=133) of the academic year 2021-22. Complete enumeration of the students was done and all willing students were enrolled. 

### Inclusion and Exclusion criteria

All the third-year (MD3) undergraduate medical students who were willing to participate and gave written informed consent were included in the study. The students who did not give their willingness or did not complete the study (pretest, active learning session, posttest, and questionnaire) were excluded from the study. 

### Study design and execution

**Figure 2:**
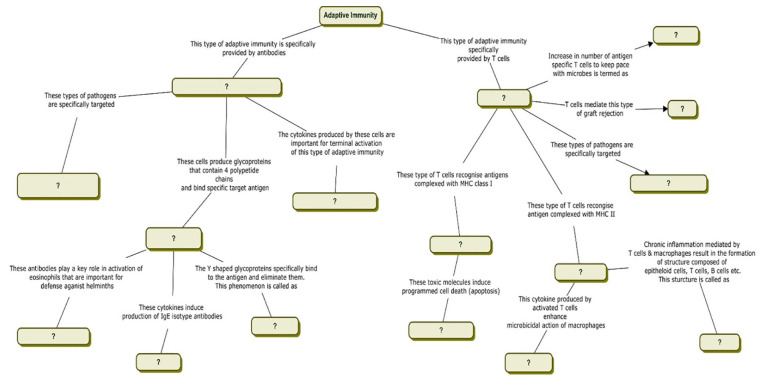
Concept map on Adaptive immunity as an example

**Figure 3:**
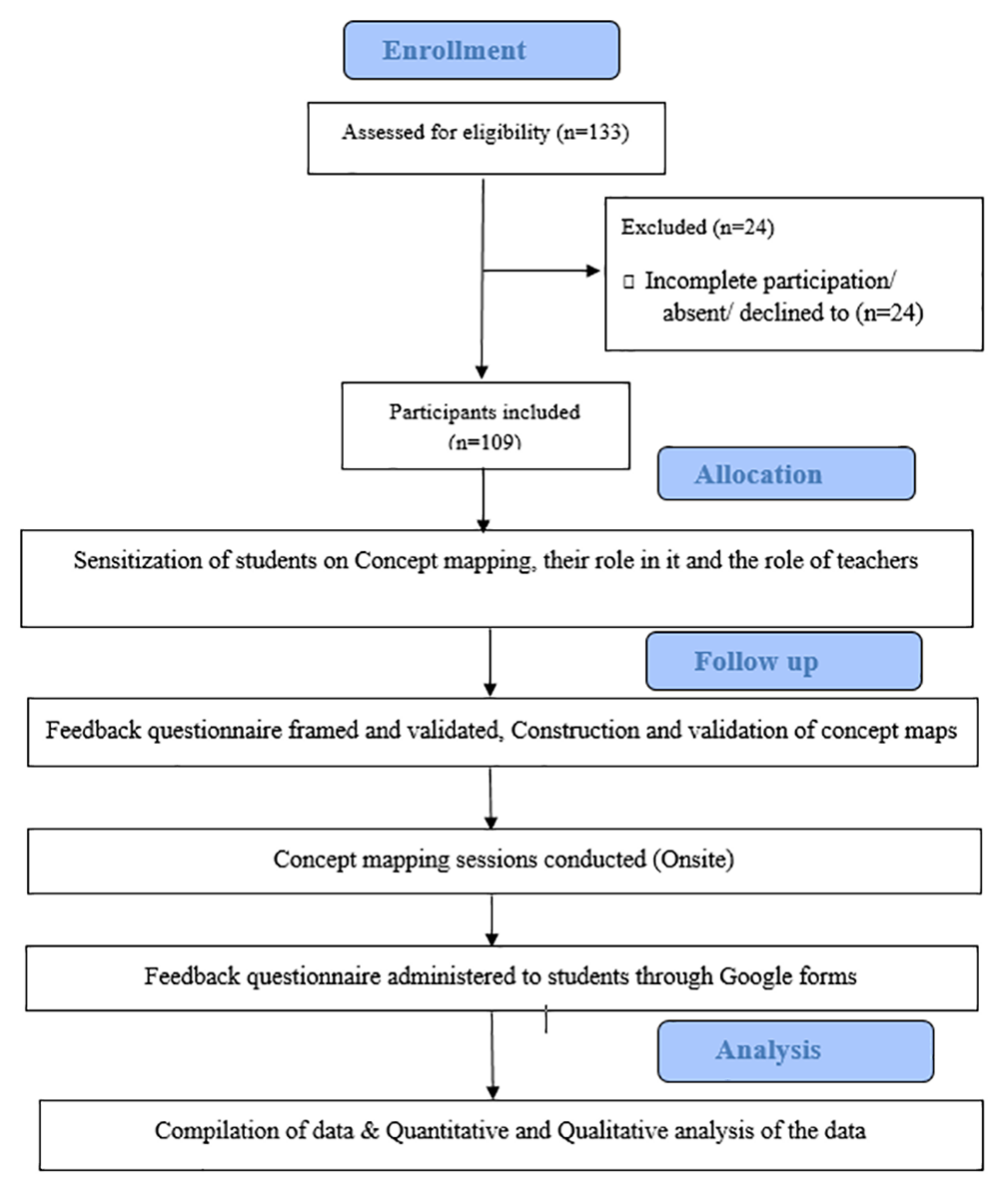
Flow chart of a study design

Concept maps on different topics of immunology such as innate, humoral, and cellular immune responses, the complement system, cytokines, and T cell and B cell development were designed by using Concept map (C-map) application developed by the Florida Institute for Human and Machine Cognition (IHMC) ( 13 ). The designed concept maps were reviewed by the immunology faculty members and experts for reliability and feasibility. Figure 2 represents an example of a concept map. A session was held to orient all the participants with an aim to develop understanding of the concept map tool. Figure 3 represents the flow chart of the study design. The whole batch was divided into 3 groups with 44-45 students in each group and separate sessions were held for each group during regular scheduled small group activity sessions of 2-hour duration. During each session, the students were randomly divided into a team of 3-4 members and for each team, predesigned concept maps were distributed to discuss critically and link the different concepts with words or phrases. After all teams completed the task and were ready with the answers, each item in the concept map was discussed in detail by the faculty members. All doubts and misconceptions were cleared with additional feedback. At the end of the immunology course after completing all the sessions, participants’ perception on utilization of concept maps as a tool for promoting their learning process was assessed. A pre-designed, pre-validated self-administered questionnaire with items aligned with a research question was used for the evaluation. The questionnaire was validated by subject experts for relevance, clarity, necessity, and feasibility. The final questionnaire included 10 quantitative items on 3 point Likert Scale (agree, neutral, and disagree), and one open-ended question (qualitative) to receive the students’ comments on concept map technique as a teaching-learning strategy. The questionnaire was sent through a Google feedback form. The result of Cronbach’s alpha test done with the SPSS version 22 was calculated as 0.936, thereby representing the reliability of the questionnaire. 

### Statistical analysis

The responses were collected, cleaned, entered to Microsoft Excel, and analyzed using SPSS software version 22. The results obtained were tabulated in the form of numbers and percentages. The 3-point 10-item questionnaire was analyzed to represent the perception of the participants regarding the effectiveness of concept map-based learning strategy in encouraging their lifelong learning process and was tabulated as frequencies and proportions as Agree (%), Neutral (%), and Disagree (%). The opinions regarding the teaching method was analyzed qualitatively for representative quotations until saturation of ideas was achieved. Quantitative data results were prioritized but qualitative data was used to support or enhance the quantitative data. 

## Results

Out of 133 eligible participants, 109 students completed the study and were included in the study. The perception of the participants regarding the effectiveness of concept map-based strategy in encouraging their lifelong learning process is displayed in Table 1.

**Table 1:** Students’ perception on utilization of concept-maps as a tool in Immunology course

Questionnaire item	Agree (%)	Neutral (%)	Disagree (%)
Constructed map were clear and well understood.	91 (83.5)	11 (10.1)	7 (6.4)
Concept maps enhanced my motivation to learn.	89 (81.7)	11 (10.1)	9 (8.3)
Concept maps helped to understand difficult topics more easily.	78 (71.6)	23 (21.1)	8 (7.3)
Concept maps enhanced meaningful learning and deeper understanding.	90 (82.6)	11 (10.1)	8 (7.3)
New information in concept maps well correlated to already known.	84 (77.1)	15 (13.8)	10 (9.2)
Concept maps improved my reasoning skills.	89 (81.7)	11 (10.1)	9 (8.3)
Concept maps improved my critical thinking.	91 (83.5)	12 (11.0)	6 (5.5)
Studying topic with concept maps is enjoyable and more interesting.	88 (80.7)	8 (7.3)	13 (11.9)
Concept maps promoted my discussion with peers.	87 (79.8)	10 (9.2)	12 (11.0)
I feel more topics can be taught in other microbiology topics.	81 (74.3)	18 (16.5)	10 (9.2)

The data reveals that approximately 80% of the students appreciated concept mapping as an effective tool for enhancing their lifelong learning skills. Four out of five students opined the constructed concept maps were clear and easily understandable, and the innovative concept map approach was enjoyable and more interesting. Majority of the students (>80%) agreed that concept maps motivated them to learn, promoted discussion with peers, enhanced reasoning skills, critical thinking, deep understanding, and thus facilitated meaningful learning. Nearly 3 out 4 participants suggested including concept maps for microbiology topics. Additionally, the participants were asked to write one entity that they liked the most in the concept map session. 

### Students’ feedback to open-ended question

Most of the students expressed that the concept map teaching-learning strategy motivated them to learn and enhanced their understanding of concepts in an easy and simplified manner. Many students accepted that concept maps helped them to link existing knowledge to the new information effectively. A number of students felt that concept maps improved their critical thinking and problem-solving ability. Further, majority of the students indicated that concept maps allowed them to correct their misconceptions, revise the topic efficiently, and thus facilitated long term retention of knowledge of the topic. A representative sample of the students’ responses is given in Table 2.

**Table 2:** General Comments/suggestions by students on concept map teaching-learning strategy

S. no.	Selected students’ comments
1	Very good way of learning how to connect different concepts.
2	It gives a better perspective of the topic when you are looking at all of the main points at the same time and make the points more related to each other.
3	It made me think a lot which enhanced my concepts of the topic. It even gave me a clearer picture of how the topic is like.
4	Discuss with friends and share the information also link and combine the concept to better understanding.
5	Understand how different concepts are linked to each other and helps us in connecting the ideas and information together.
6	Revise the material that we studied along with few new information.
7	Make it easier to study, gather information, and revise topic.
8	Better way of studying and remembering key points of the topic.
9	It enhances critical thinking and connecting things well.
10	It benefited me in understanding key concepts of the lectures, I enjoyed it.
11	It helped to link new knowledge with old knowledge and think deeply to understand the topic well.
12	Made me communicate with students who are new to me. Discussion strengthened my knowledge about the topic.
13	Better way of studying and remembering key points of the topic, increases knowledge retention.

## Discussion

In modern competency-based medical education, there is a considerable need for the medical educators to groom future doctors so that they can analyze and solve diverse problems and tackle any given situation in modern medicine ( 22 ). This demands a shift from teacher-centered learning strategies to student-centered innovative learning methods where students are allowed to discuss, think critically, and take rational decisions related to patient care. Students are required to exercise, debate among themselves, and scrutinize the given solutions during any problem-solving approach which, in turn, will allow them to make appropriate decisions. Concept map approach is an innovative educational strategy, well appreciated by medical educators to foster both learners’ knowledge integration as well as self-assessment ( 14 , 20 ). 

The aim of our study was to introduce a concept map-based teaching-learning strategy in the immunology course and to know the perception of the students towards it in achieving the multiple learning objectives of the cognitive domain. The study exhibited a positive perception from the majority of students (> 80%) regarding the use of concept map-based teaching-learning strategy in immunology. Nearly four out of five students expressed that they felt fresh, relaxed, and enjoyed the concept map sessions, with more concentration and motivation to learn. The majority (71.6%) acknowledged that concept maps helped them to understand difficult topics more easily. Further, 77.1% indicated that concept maps were useful to establish the correlation between new information and the already existing knowledge. Furthermore, concept maps allow students to know how ideas are connected and organized in a meaningful way, and thus promotes their deeper understanding of the topic. These findings are in coherence with the results of studies conducted elsewhere ( 14 , 23 , 24 ). 

Improving medical students’ ability to think critically, explore varied possibilities, and relating the information gathered to solve the problem is one of the missions of modern medical education. Therefore, it is the responsibility of medical educators to teach new concepts through innovative strategies to achieve these objectives. In our study, majority of students (nearly 80%) indicated that the concept map-based method enhanced their critical thinking and problem-solving ability. Maryam, *et al*. in their concept map study demonstrated significant improvement in critical thinking skills among the students in the experimental group compared to the control group ( 11 ). Furthermore, to enhance lifelong learning, students had to work as a team, communicate with team members, accept or argue in a friendly manner with others, and arrive at the final creation. By this, students develop skills relating to professional interactions and teamwork. Sleiman, *et al*. in their study on case-based learning by using concept map assessed all these objectives among medical students during the pre-clinical period through different rubrics throughout the course ( 8 ). In another similar study, students perceived that concept map learning strategy enhanced their understanding of the subject and retention of knowledge for a longer duration of time, thus facilitating lifelong learning ( 14 ). In congruent with this, the majority of our participants agreed that concept map was an effective tool to encourage peer discussion, teamwork, deeper understanding of the topic, greater knowledge retention, and mold them as better lifelong learners. Finally, 3 out 4 participants opined that concept map-based learning strategy should be incorporated to more topics in immunology, indicating it had a positive impact on promoting lifelong learning skills. This can be ascribed to students’ active participation in the learning process during the session. The graphical organization and representation of information in a concise and well-organized manner in concept maps enhances their curiosity, collaborative thinking, and thus enhances their deeper understanding and meaningful learning ( 14 ). When the students were asked to comment on effectiveness of utilizing concept maps as a teaching-learning strategy, the majority penned down positively. The students’ comments indicate that concept maps facilitate their metacognitive skills. This finding is consistent with the results of the study conducted by Martin-Dunlop, *et al*. ( 25 ). 

### Limitation

Our study had few limitations. Firstly, to align the study style of our students, we designed concept maps with linking words or phrases. However, studies have shown students become better lifelong learners if they construct concept maps on their own. Secondly, the study only assessed the students’ perception on the effectiveness of concept map-based strategy to nurture them as lifelong learners. It would have been better if effectiveness was measured by comparing the students’ exam grades on the topics taught by concept map compared to topics taught without the concept map strategy. Finally, the study was a single centric study involving a group of MD3 cohorts of CoMHS. Hence the results of the study cannot be generalized and warrant more multi-centric studies for transparency of results.

## Conclusion

From the present study, it can be concluded that concept maps are innovative learning strategies, which play a significant role in helping learners to understand complex problems in a much better way. They promote metacognition in terms of critical thinking, problem solving, motivation, professional interaction, peer discussion, and better knowledge acquisition. Furthermore, concept maps facilitate designing a unified curriculum to promote lifelong learning skills among students. Overall, concept maps are well suited for promoting students’ comprehension of the learning material and enhancing their understanding of new material. 

## Acknowledgement

Authors are indebted to third-year (MD3) undergraduate medical students of the academic year 2021-22 for their voluntary participation and helping us to complete the study. 

## Authors' contribution

M.B.S, V.N, R.A contributed to the conception and design of the work; the acquisition, analysis, or interpretation of data for the work. All Authors contributed in drafting and revising the manuscript critically for important intellectual content. All authors have read and approved the final manuscript and agree to be accountable for all aspects of the work in ensuring that questions related to the accuracy or integrity of any part of the work are appropriately investigated and resolved.

## Conflict of Interest:

None declared. 
